# Shaping linguistic input in parent‐infant interactions: The influence of the Infant's temperament

**DOI:** 10.1111/infa.12629

**Published:** 2024-10-01

**Authors:** Antonia Götz, Eylem Altuntas, Marina Kalashnikova, Catherine Best, Denis Burnham

**Affiliations:** ^1^ The MARCS Institute for Brain, Behaviour and Development Western Sydney University Sydney New South Wales Australia; ^2^ Basque Center on Cognition, Brain and Language San Sebastian Spain; ^3^ IKERBASQUE Basque Foundation of Science Bilbao Spain

## Abstract

Parent‐infant interactions highlight the role of parental input, considering both the quality, infant‐directed speech, and quantity of interactions, adult words and communicative turns, in these interactions. However, communication is bidirectional, yet little is known about the infant's role in these interactions. This study (*n* = 35 4‐month‐old infants) explores how infant‐directed speech, the number of adult words and turn‐taking (both measured by the LENA system) are correlated with infants' temperament. Our findings reveal that, while mothers use the typical characteristics of infant‐directed speech, they are not correlated with the infant's temperament. However, we observe more adult‐infant turn‐taking in both introverted infants (with lower Surgency scores) and infants with lower attention regulation (with lower Regulatory/Orienting scores). The number of adult words was not correlated with infants' temperament. We suggest that infants with an introverted temperament prefer quieter exchanges that may lead to more turns and that infants with lower attention regulation might create more opportunities for interactions due to their lower level of self‐regulation. These findings suggest that infants' temperament is associated with how adults talk *with* infants (communicative turns) rather than how adults talk *to* infants (infant‐directed speech, number of adult words). Our results underscore the infant's role in parent‐infant communication.

## 1 INTRODUCTION

Social interaction and communication are intricate processes that are the cornerstone of human relationships, enabling the exchange of information, ideas, and emotions among individuals. Research with adults shows that communication is a dynamic exchange involving at least two individuals who convey information through both verbal and non‐verbal behaviors. Each individual brings their own personality into the communicative situation, and these influence the dynamics of the interaction. For example, a more extraverted individual tends to initiate more conversations (Frederickx & Hofmans, 2014) and articulates a greater number of words (Mehl et al., 2006). Yet, we do not know how an infant's personality affects parent‐infant interactions.

In infancy, social interaction and communication are not only crucial for meeting basic needs but also serve as essential foundations for cognitive (Tomasello, 1995), emotional (Izard et al., 2011), and social (Ainsworth et al., 2013) development. However, research on parent‐child interactions has emphasised the role of parents in child development, focusing on parental responsiveness in terms of both the quality and quantity of interactions and also the link to subsequent language development (Bornstein & Tamis‐LeMonda, 1989; Kalashnikova & Burnham, 2018; Tamis‐LeMonda et al., 2001). In infancy, temperament serves as a foundation for later personality and refers to behavioral and emotional reactivity (Beekman et al., 2015). In the current study, we examine the influence of an infant's temperament on parent‐infant interactions, as indexed by the use of infant‐directed speech (IDS) by mothers, the quantity of adult words that infants encounter, the number of communicative turns between mother and infant, and the number of vocalisations by the infant.

IDS differs significantly from adult‐directed speech (ADS) most notably in its acoustic and phonetic characteristics, viz., (i) acoustically, IDS has a higher mean fundamental frequency (f0) and more pronounced variations in f0 compared to ADS, and (ii) phonetically IDS involves modification of the F1/F2 vowel space as measured by the area of the triangle described by the three corner vowels/i, a, u/, with reliable evidence for expanded F1/F2 vowel space area in IDS compared with ADS (Cox et al., 2022). It has been shown that maternal vowel space expansion correlates with infants' speech contrast discrimination abilities (Kalashnikova & Carreiras, 2022; Liu et al., 2003) and predicts vocabulary growth beyond the first year (Kalashnikova & Burnham, 2018; Kuhl, 2004). A few studies on IDS already suggest that infants’ experiences alter early linguistic input and the pattern of maternal IDS (Kalashnikova et al., 2018; Lam & Kitamura, 2012). Lam and Kitamura (2012) conducted an innovative experiment in which they recorded mothers' IDS using a dual video setup. In this, the infant and the mother are seated in separate rooms but remain connected via a live video stream. The authors manipulated what the infant could hear by altering the volume of the mother's voice to the infant from full volume to half to zero volume. It was found that at full volume, mothers expanded their vowel space (compared to that in adult‐directed speech); at half volume, their vowel space expansion was diminished, and at zero volume, there was no expansion of vowel space. This effect persisted across two conditions—when mothers were aware versus not aware that their child could not hear them. This shows that when infants can hear their mother talking to them, they provide some sort of signal(s) that influence her speech, but when they cannot hear the mother, they do not provide such signals. Similarly, Kalashnikova et al. (2018) recorded mothers speaking to their infants who were at developmental risk for dyslexia and to infants who did not have a familial risk for dyslexia and found that in their IDS, mothers of not‐at‐risk infants expanded their vowel space, whereas mother of at‐risk infants did not. In a subsequent study using a novel cross‐dyad design, Kalashnikova et al. (2020) studied the speech of mothers of either at‐risk or not‐at‐risk infants to their own infant, and to two other infants, one of the same risk status as their own infant, and another of the opposite risk status in conditions in which they did not know the risk status of these other two infants. Mothers of not‐at‐risk infants expanded their vowel space when communicating with another not‐at‐risk infant but did not expand their vowel space with another at‐risk infant, whereas mothers of at‐risk infants did not make any adjustment when speaking with not‐at‐risk infants, that is, they increased their vowel space area to both their own and the other infant. These studies suggest that there are specific signals provided by not‐at‐risk infants, but not by at‐risk infants, which appear to elicit maternal vowel space expansion in IDS. Mothers of at‐risk infants' failure to adjust their speech from at‐risk to not at‐risk infants show that by virtue of their interaction with their own at‐risk infant, they were less sensitive to whatever “want‐vowel‐hyperarticulation” cues the not at‐risk infants displayed. These results show that there are both definite contributions of infants' cues to parent‐infant interaction as well as effects of parental experience.

Another fundamental aspect of communicative interactions is turn‐taking in vocal interactions. The importance of vocal turn‐taking for children's linguistic development has recently been highlighted in studies focusing on the effects of parental coaching on children's vocabulary growth. For example, Ferjan Ramírez et al. (2019, 2020) have found that increases in vocal turn‐taking and characteristics of infant‐directed speech during parental coaching from 6 to 18 months correlate with infants' vocal activity and early word production by 18 months of age, as well as vocabulary size and growth from 18 to 30 months (Huber et al., 2023). Other studies have shown that infants of mothers with high anxiety levels engage in fewer turns in vocal interactions and have lower vocabulary scores compared to infants of mothers with low anxiety levels (Brookman et al., 2020). A recent meta‐analysis by Wang et al. (2020) highlights that number of turns taken between parent the infant, and number of children's vocalisations have a greater impact on children's later language skills than the number of adult words spoken to the infant.

The key characteristic of communicative turn‐taking is that infants are involved in social interaction with caregivers in a dynamic and reciprocal manner. This interaction involves a sequence of communicative exchanges in which both the infant and caregiver take turns contributing to the vocal exchange. During these exchanges, infants not only respond to the vocalisations of the caregiver but also initiate their own communicative signals. The process of communicative turn‐taking is marked by mutual attention and the synchronisation of behaviors, creating a shared communicative space (Bruner, 1985; Tomasello, 1995). Consequently, communicative turn‐taking between infants and caregivers has been proposed to be the groundwork for infants' linguistic, cognitive and social development (e.g., Dunham & Dunham, 1995; Ferjan Ramírez et al., 2020; Golinkoff et al., 2019; Hilbrink et al., 2015).

Moreover, research investigating the interactions between parents and infants has demonstrated that the way in which parents talk to their babies relies on a bidirectional relationship between infants and parents. For instance, Donnelly and Kidd (2021) collected daylong recordings every 3 months of infants longitudinally between 9 and 24 months. Their results show that the infants' vocabulary grows as the infants increase their turn‐taking and, reciprocally, their turn‐taking increases as their vocabulary grows. Dailey and Bergelson (2023) provide additional evidence supporting the bidirectional relationship between infants and parents in their communication patterns. They recorded the amount of language input to infants aged 7–17 months and found that parents (of both boys and girls) increased their language input precisely when infants began to utter their first words. In essence, the greater the child's vocalisations, the more words parents will produce in interactions with the child.

Taken together, these studies suggest that the driving force in infant‐parent interaction is the behavioral feedback from the infant, which guides vowel space expansion (Kalashnikova et al., 2018; Lam & Kitamura, 2012), the growth in turn‐taking and vocabulary development (Donnelly & Kidd, 2021), and the amount of input that the infant receives (Dailey & Bergelson, 2023). Thus, parents respond to their infants' linguistic and cognitive needs to communicate successfully (see also Leung et al., 2021). While existing research points to the significant impact of behavioral feedback from infants on parent‐infant interactions, it leaves an unexplored dimension: the role of infant temperament. Understanding how infants' temperament intersects with parent‐infant communication can contribute to an in‐depth understanding of the dynamics at play in these interactions.

In research with adults, it has been observed that an individual's personality is associated with their communication patterns. In a study in which adults wore mobile recording devices to capture their social interactions, it was found that extraversion and emotional stability, in particular, were positively associated with a greater number of words spoken and more conversations (Mehl et al., 2006). This demonstrates that adults' personality is associated with their conversational behavior. This begs the question of whether infants' temperament, the precursor of personality, is associated with vocal aspects of parent‐infant interactions. The current study addresses the unresolved question of how the infant's personality might correlate with parental‐infant interactions. It has been shown that the infants' temperament influences various aspects of their lives. For instance, temperament is associated with maternal tendencies to use food as a calming mechanism for the infant (Schneider‐Worthington et al., 2022), maternal and paternal internalising symptoms (Spry et al., 2020) and has also been shown to have an influence on linguistic development (Bruce et al., 2022; Dixon Jr & Smith, 2000; Morales et al., 2000). The present study aims to address the following questions: (1) Is maternal vowel space expansion correlated with infant temperament? (2) Does the quantity of adult words infants are exposed to correlate with the infants' temperament? (3) Does the number of infant‐parent communicative turns and/or the number of infant vocalisations correlate with infant temperament?

With respect to the first of these three questions, we might expect that maternal vowel space expansion is not correlated with the infants' temperament, as previous studies suggest that the quantity of adults' vocalisations to their infants is more correlated with the infant's personality than is the quality of the adults' speech. However, given that mothers show vowel space expansion based on infant characteristics (Kalashnikova et al., 2018, 2020), we might also expect that mothers expand their vowel space in relation to the infant's temperament. Consequently, both hypotheses seem plausible in the light of the current study. Regarding the second question, if temperament influences parent‐infant interactions, we expect adults to use speech more frequently to comfort and soothe infants with lower emotional self‐regulation. Consequently, we would anticipate a higher number of adult words directed toward these infants. Finally, turning to the third question, (if temperament has a role to play in parent‐infant interactions, then we would expect that infants displaying higher levels of extraversion traits would engage in more turn‐taking). The present study was conducted according to guidelines laid down in the Declaration of Helsinki, with written informed consent obtained from a parent or guardian for each child before any assessment or data collection. All procedures involving human subjects in this study were approved by the Human Ethics Committee at the Western Sydney University. With their parents and show a greater number of vocalisations.

In early infancy, temperament can be measured with the Infant Behavior Questionnaire‐Revised (IBQ‐R; Gartstein & Rothbart, 2003). The IBQ‐R measures infant temperament on three dimensions: Surgency, Negative Affect, and Regulatory/Orienting (Putnam et al., 2014). Surgency, akin to extraversion in personality, pertains to infants exhibiting higher activity levels and a proclivity for novelty‐seeking. Negative Affect relates to an infant's capacity to regulate their emotional states. Lastly, Regulatory/Orienting includes abilities such as sustaining attention and resisting distractions (Gartstein & Rothbart, 2003; Putnam et al., 2014).

One study has directly investigated the connection between infants' temperament and maternal input. Spinelli et al. (2018) explored the association between the quality of input and the infant's temperament. Temperament was measured at 3 months with the IBQ‐R. Maternal input was assessed based on lexical variability (such as the frequency of word types) and syntactic complexity (measured by the mean length of maternal utterances) during interactions with infants at 6, 9, and 12 months of age. It was found that language production, demonstrated by expressive vocabulary at 18 and syntactic complexity at 24 months, was moderated by maternal input at 6 and 9 months and the infant's duration of orienting abilities at 3 months. The connection between infants' orienting abilities at 3 months, maternal input, and subsequent language skills suggests that infants with stronger orienting abilities and mothers who employed more syntactically complex and lexically diverse language tended to exhibit greater language production skills. However, no correlation was found between infants' temperament and language outcomes for mothers who used less linguistically complex and varied language.

It is worth noting that the Spinelli et al. (2018) study primarily focuses on maternal input quality, that is, lexical variability and syntactic complexity. However, while IDS research suggests that infants influence the *quality* of parental input, they also influence the *quantity*, the amount, of parental input they are exposed to in their everyday routines. Recently Dailey and Bergelson (2023) proposed that infants play a significant role in shaping the *quantity* of language input. However, no research has explored the relationship between the quality and quantity factors of maternal input and the influence of infants' temperament on it. Subsequently, the question of the current study revolves around whether the temperament of the infant impacts the quality of parental input, as indicated by the vowel space expansion, or the quantity aspects, indicated by the number of adult words, communicative turns and infant vocalisations.

### 1.1 Current study

The aim of this study is to investigate the nature of the relationship, if any, between infant temperament and the linguistic input from their mothers. Quality of linguistic input was assessed by the phonetic characteristics of IDS, recorded in an in‐lab play session. Quantity was assessed by a daylong recording session using the Language Environment Analysis (LENA) system (e.g., Greenwood et al., 2018; Weisleder & Fernald, 2013), which provides tallies of the counts of adult words, child vocalisations, and communicative turns (Ganek & Eriks‐Brophy, 2018). Please note that we use the term communicative turns instead of conversational turns, as a conversation typically involves verbal exchanges of thoughts or information, which non‐verbal infants cannot fully participate in. To measure infant temperament, we used the IBQ–R, a robust questionnaire that loads onto three factors: I. Surgency, II. Negative Affectivity, and III. Regulatory/Orienting (Putnam et al., 2014).

Based on previous studies, we predict that infants with a more extraverted personality (higher scores in Surgency) will have more turns and more vocalisations. Infants with a lower capacity for emotional self‐regulation (operationalised by Negative Affectivity) are likely to elicit an increased number of adult words, as adults may use speech as a means to comfort and soothe the infant. With respect to the association between the infant's temperament and maternal vowel space expansion, two alternative hypotheses seem plausible: on one hand, the infant's temperament may not be correlated with maternal vowel space expansion given that personality has been found to be associated with quantity rather than quality aspects of speech (Mehl et al., 2006); on the other hand, the infant's temperament may be correlated with maternal vowel space expansion given that temperament has found to correlate with the complexity of maternal syntactic and lexical speech (Spinelli et al., 2018) and mothers' vowel space expansion has been found to correlate with other infant characteristics, such as infants' hearing abilities and at‐risk status for dyslexia (Kalashnikova et al., 2020; Lam & Kitamura, 2012).

## 2 METHOD

### 2.1 Participants

Thirty‐five 4‐month‐old infants (*M*_age_ = 4.41 months, SD = 0.64, 16 females, 19 males) participated in this study. The sample size was estimated based on estimated effect sizes for maternal vowel space expansion in IDS compared to ADS. For an effect size of 0.8 (based on the lower value on vowel space expansion for Australian English, see meta‐analysis by Cox et al. (2022) and 90% power, the estimated sample size is 33 infants. These infants were raised exclusively in an English‐speaking environment (max. of 4 h/week exposure to another language), had normal hearing, and had no reported neurological conditions or learning/language impairments. They were recruited from the local BabyLAB register, and their participation was approved by the university's ethics committee. The majority of mothers completed an undergraduate degree or higher (PhD = 2, completed masters = 8, completed undergraduate studies = 13, completed college or technical degree = 7, completed high school = 5). Of the participating families, parents reported that infants had no siblings (*n* = 28), one other sibling (*n* = 6) or two other siblings (*n* = 1). The present study was conducted according to guidelines laid down in the Declaration of Helsinki, with written informed consent obtained from a parent or guardian for each child before any assessment or data collection. All procedures involving human subjects in this study were approved by the Human Ethics Committee at the Western Sydney University. Parents received a $20 travel reimbursement, and the infants received a gift and a graduation certificate upon completion of the study.

### 2.2 Procedure

The procedure consisted of one lab play session and one at‐home session. During the lab session, each parent was recorded while interacting with their child to measure the acoustic characteristics of IDS. After this session, parents received a LENA device and instructions on its use and were asked to record at least 10 h of the interactions in the child's environment on a day they felt comfortable. Parents reported that they chose mostly at home with occasional short outings to a park was often chosen. The at‐home recording occurred 7–10 days after the lab visit. During this period, parents were also given a temperament questionnaire to complete. All parents in the current study participated in the lab session, the daylong recording session and all filled in the temperament questionnaire.

#### 2.2.1 Infant‐directed‐speech

For the IDS recording, mothers and infants were assigned to a single room. Mothers and their infants sat on a soft carpet on the floor. All interactions were recorded on audio and video. For the video recordings, we used four cameras to capture all angles of the mother‐infant interaction. The audio was captured using two microphones; one was an over‐the‐ear microphone worn by the mother and directed to the mother's mouth to capture the mother's speech directly, and another microphone was placed above the mother‐infant interaction to capture the mother, infant and ambient sound. Only the audio from the microphone worn by the mother was analyzed in this study.

The mothers were provided with five toy objects to prompt elicitations of multiple productions of the target words ‘sheep’, ‘shoes’, and ‘shark’ with the target vowels/i/,/u/and/a/. Before the IDS sessions, mothers were presented with video‐recorded instructions spoken by a native speaker of Australian English to ensure that they were introduced to the target words with Australian English pronunciation. The instructions included directions for the mothers to play naturally with their infant by using the three target‐named objects and to aim to use each word multiple times. Immediately following the infant‐directed speech (IDS) recording sessions, the experimenter interviewed the mother in the same set‐up to elicit adult‐directed speech (ADS) productions of the target words in communication with another adult. The IDS and ADS sessions lasted between 10 and 15 min in total.

##### Acoustic measures and data analysis

We used the Praat software (version 6.1.36, Boersma & Weenink, 2022) to identify, segment and perform the acoustic analysis of the target words. In total, we identified 2440 segments with the target words (1569 IDS and 871 ADS). An additional 111 segments were excluded from the analysis because the segment included noise (e.g., interferences from the infant), or because the formants were not identifiable due to creaky voice or whispering. Praat scripts were used to measure the formants (F1, F2) of each target vowel (/i/,/u/,/a/) using the mean value in Hz from 40% to 80% points of each vowel's duration (Munhall et al., 2009). Furthermore, the maximum formant search was set to 5500 Hz with an applied pre‐emphasis to frequencies above 50 Hz.

#### 2.2.2 Day‐long recording

The daylong recordings were obtained and analyzed using the LENA system (Gilkerson & Richards, 2008), which is a portable audio recorder installed in a pocket on a vest worn by the baby to record speech sounds and vocalisations in the infant's environment. Completed recordings are uploaded and processed via machine‐learning software (Xu et al., 2009). LENA categorises auditory information into speech or non‐speech (such as television/electronic sounds, noise, and silence) (Ganek & Eriks‐Brophy, 2018), and within the speech category, again via machine learning, adult or infant speech.

Parents were taught how to operate LENA and given the device to take home. At home, the parents switched on LENA in the morning and placed it inside the vest, which was worn by the infant throughout the day. During the day, the vest was only to be removed when the infants slept or needed to be cleaned or bathed, but the LENA device was left on so to allow continued recording from nearby.

When the LENA device was returned to the lab, the recordings were processed using LENA software. The mean length of recordings was 11.34 h (range 9.31–14.04 h). More importantly, three scores were extracted—Adult Word Count (AWC), Child Vocalisation Count (CVC), and Communicative Turn Count (CTC). AWC is the number of adult words that are spoken to or near the infant. CVC is the number of speech‐like vocalisations by the infant. CTCs are the number of alternations between infant and adult vocalisations, with a response from either communication partner within 5 s. In both the CVC and CTC indices, crying and other vegetative sounds from the infant do not contribute to the counts.

#### 2.2.3 Temperament questionnaire

The IBQ‐R short form consists of 91 items (Putnam et al., 2014). In each item, caregivers are asked to assess their infant's behavior in different everyday situations based on the past 2 weeks. Each item is scored through a Likert‐type scale from 1 (never) to 7 (always). The IBQ‐R measures Surgency (such as laughter: “When tossed around playfully, how often did the baby laugh?”), Negative Affectivity (such as fear, sadness: “At the end of an exciting day, how often did your baby become tearful?”) and Regulatory/Orienting (such as low‐intensity pleasure, duration of orienting: “How often during the last week did the baby play with one toy or object for 5–10 min?”). The questionnaires were completed by the parents (usually the mothers) when the infants were 4 months of age.

## 3 RESULTS

### 3.1 Infant‐directed speech

The results from the acoustic analyses (formant values, number of segments) for each vowel and register are provided in Table 1. To calculate the vowel space, we used the phonR package (McCloy & McCloy, 2016). Based on our research questions, we calculated two vowel spaces (one for IDS and another for ADS) for each participant. To calculate whether mothers expanded their vowel space in IDS relatively compared to ADS, we derived hyper‐vowel scores in which the vowel space area in each mother's IDS is divided by the vowel space area in her ADS (see also Kalashnikova & Burnham, 2018; Kalashnikova & Carreiras, 2022). This score has the advantage of each mother's ADS acting as a within‐speaker control. A score greater than one indicates an expansion of the IDS vowel space compared to ADS, whereas a score less than one indicates a vowel space reduction in IDS compared to ADS. The mean vowel hyper‐score was 3.05 (range = 0.01–7.49), indicating that mothers expanded their vowel space in IDS compared to ADS (one‐sample *t*‐test against 1, *t* (34) = 2.420, *p* = 0.021).

**TABLE 1:** Number of segments, first formant (F1) and second formant (F2).

ADS				IDS		
Vowel	*n*	F1	F2	*n*	F1	F2
/i/	275	601.41 (134.71)	1709.80 (357.93)	542	650.97 (180.59)	1876.84 (313.12)
/u/	245	593.01 (134.08)	1626.70 (305.18)	467	604.65 (123.35)	1690.82 (247.66)
/a/	351	721.06 (112.99)	1542.54 (148.53)	560	698.45 (93.10)	1492.53 (126.90)

*Note*: The table displays the number of segments and results from the acoustic measures of the formants per vowel and register. Formant values are shown as the mean results in Hz with their standard deviation in brackets.

### 3.2 Validity of the daylong recording

Some studies suggest that the LENA system may either overestimate (Ferjan Ramírez et al., 2021) or underestimate (Cristia et al., 2020) the number of child vocalisations. To address this issue, we manually coded randomly selected 5‐min intervals from each daylong recording to compare the manually assessed values of AWC, CVC, and CTC against the automatically extracted values from the LENA system. For the manual coding, we applied the same rules as described in the LENA's technical report (Gilkerson & Richards, 2008). For these 5‐min intervals the mean values for the manually‐coded versus LENA‐coded were: for AWC manual 130.32 (SD = 183.52), LENA 156.69 (SD = 194.84); for CVC manual 6.48 (SD = 11.60), LENA 9.79 (SD = 16.96); and for CTC manual 2.77 (SD = 4.26), LENA 2.58 (SD = 2.88). Pearson's product‐moment correlations were conducted to assess the relationship between the values from manual coding and from LENA. The correlations were significant for all three measures: AWC (*r* (33) = 0.950, *p* < 0.001), CVC (*r* (33) = 0.790, *p* < 0.001) and CTC (*r* (33) = 0.710, *p* < 0.001). These high and significant correlations of LENA with manual coding provide evidence for external validity of the LENA coding in this context, and so we decided to use the values provided by the automatic output from the LENA system in our analyses.

### 3.3 Temperament and quality of parental input

All computations were performed using R (version 4.0.4, R Core Team, 2021). The results from the temperament questionnaire showed a mean of 4.74 (SD = 0.70) for Surgency, 3.99 (SD = 1.07) for Negative Affectivity and 5.31 (SD = 0.70) for Regularity/Orienting scores. To assess the links between a mother's quality of IDS and their infant's temperament, we used the vowel space hyper‐scores (IDS‐area/ADS‐area) along with the temperament scores. We conducted two analyses for this assessment. First, given the bidirectional nature of parent‐infant interactions, we performed a correlational analysis between maternal vowel space expansion and infants' temperament. Second, given that infants' temperament is assumed to be partly innate and stable across development (e.g., Bridgett et al., 2015; Carranza Carnicero et al., 2000; Rothbart et al., 2000), we also performed a regression analysis to investigate whether infants' temperament predicts maternal vowel space expansion.

With respect to the first, Pearson's product‐moment correlations were conducted to assess the relationship between maternal vowel space expansion and the various aspects of infant temperament. There were no significant correlations for any of the three infant aspects: Surgency (*r* (33) = 0.224, *p* = 0.272), Negative Affectivity (*r* (33) = −0.126, *p* = 0.540) and Regulatory/Orienting (*r* (33) = −0.095, *p* = 0.643).

For the regression analyses, we formed three hierarchical multiple regression analyses with Surgency, Negative Affectivity, and Regulatory/Orienting as predictors of the vowel space hyper‐score as the dependent variable. In the first step, we included the scores from the temperament questionnaire (Surgency, Negative Affectivity and Regulatory/Orienting) as predictor variables. In the second step, we added maternal education, and in the third step, we added infant gender. The results are shown in Table 2, and it can be seen that none of the temperament scales predicted the mother's vowel quality or their vowel hyper‐scores.

**TABLE 2:** Multiple regression model with vowel space area.

Predictor	*β*	SEM	*t*‐value	*p*‐value
Model 1: Vowel space area				
*R* ^2^ = 0.158, Δ*R* ^2^ = 0.038, *F* (3, 31) = 1.314, *p* = 0.296				
Regulatory/Orienting	0.415	0.345	1.201	0.243
Surgency	−0.398	0.316	−1.258	0.222
Negative affectivity	0.299	0.193	1.547	0.137
*R* ^2^ = 0.1893, Δ*R* ^2^ = 0.0271, *F* (4, 30) = 1.893, *p* = 0.355				
Regulatory/Orienting	0.375	0.350	1.070	0.298
Surgency	−0.376	0.319	−1.179	0.252
Negative affectivity	0.294	0.194	1.515	0.145
Education	0.137	0.156	0.877	0.391
*R* ^2^ = 0.194, Δ*R* ^2^ = −0.018, *F* (5, 29) = 0.209, *p* = 0.492				
Regulatory/Orienting	0.335	0.377	0.889	0.385
Surgency	−0.373	0.327	−1.143	0.267
Negative affectivity	0.294	0.199	1.478	0.156
Education	0.157	0.170	0.922	0.368
Gender	−0.169	0.498	−0.34	0.737

### 3.4 Temperament and quantity of parental input

To assess the relationship between infants' temperament and quantity measurements, we used data extracted by the LENA device. The mean values for AWC were 17,180.96 (SD = 5923.68), for CTC 267.74 (SD = 115.63), and for CVC 808.31 (SD = 442.19). As in the previous analyses, we first performed a Pearson correlation between the factors of caregiver's input and the infants’ temperament, and secondly, multiple regression analyses.

#### 3.4.1 Adult Word Count

There were no significant correlations between AWC and Surgency (*r* (33) = −0.108, *p* = 0.537), AWC and Regulatory/Orienting (*r* (33) = −0.172, *p* = 0.324) or AWC and Negative Affectivity (*r* (33) = 0.064, *p* = 0.716).

#### 3.4.2 Communicative Turn Count

**FIGURE 1:**
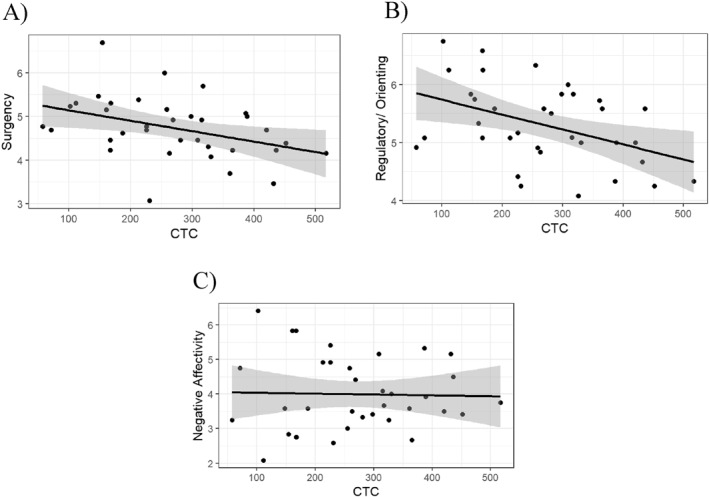
Temperament and Communicative Turn Count (CTC). (a) Surgency. (b) Regulatory/Orienting. (c) Negative Affectivity. Surgency (top left) and Regulatory/Orienting (top right) correlate significantly with communicative turns (CTC). Negative Affectivity did not correlate with CTC.

There was a statistically significant negative correlation between CTC and Surgency (*r* (33) = −0.393, *p* = 0.019), and a significant negative correlation between CTC and Regulatory/Orienting (*r* (33) = −0.428, *p* = 0.010), but no significant correlation between CTC and Negative Affectivity (*r* (33) = −0.027, *p* = 0.876). See also Figure 1.

#### 3.4.3 Child Vocalisation Count

There was no significant negative correlation between CVC and Surgency (*r* (33) = −0.329, *p* = 0.053) Regulatory/Orienting (*r* (33) = −0.310, *p* = 0.070), or CVC and Negative Affectivity (*r* (33) = 0.041, *p* = 0.815).

As an exploratory analysis, we examined the relationship between communicative turn initiation (by adults vs. infants) and infants' temperament. Since the automatic measurements from the LENA system have not been validated for identifying the initiation of turns, we relied on the outcomes from the 5‐min intervals obtained through manual coding. Using manual coding, we defined a turn initiation as the first vocalisation by either the infant or an adult within a 5‐s window. For instance, if the adult spoke and the infant vocalised within 5 s, the turn was considered initiated by the adult. If the infant vocalised after 5 or more seconds, the interaction was not counted as a communicative turn. Conversely, if the adult responded within 5 s to the infant's vocalisation, the interaction was counted as a communicative turn initiated by the infant. Based on these rules, we found that 67.1% of the turns were initiated by the adult, and 32.9% by the infant. In an exploratory analysis, we correlated the data with the infant's temperament scores. Neither the initiation by the adult (Surgency: *r* = −0.03, Negative Affectivity: *r* = 0.20, Regulatory/Orienting: *r* = −0.10) nor by the infant (Surgency: *r* = −0.04, Negative Affectivity: *r* = 0.04, Regulatory/Orienting: *r* = −0.04) correlated with the infants' temperament scores.

In addition to the correlations, we performed three hierarchical multiple regression analyses, one each for the three LENA‐derived scores—AWC, CTC, and CVC. As in the previous section, in step 1, we included the scores from the temperament questionnaire (Surgency, Negative Affectivity and Regulatory/Orienting) as predictor variables. In the second step, we added maternal education, and in the third step, we added infant gender. The output from the Model 1 (AWC), Model 2 (CTC), and Model 3 (CVC) are displayed in Table 3.

**TABLE 3:** Multiple regression models with adult word count (Model 1), communicative turn count, (Model 2), and child vocalisation count (Model 3).

Predictor	*β*	SEM	*t*‐value	*p*‐value
Model 1: AWC				
*R* ^2^ = 0.032, Δ*R* ^2^ = −0.061, *F* (3, 31) = 0.344, *p* = 0.794				
Regulatory/Orienting	−1259.200	1710.600	−0.736	0.467
Surgency	−330.100	1677.300	−0.197	0.845
Negative affectivity	231.100	991.700	0.233	0.817
*R* ^2^ = 0.035, Δ*R* ^2^ = −0.094, *F* (4, 30) = 0.270, *p* = 0.895				
Regulatory/Orienting	−1260.800	1736.700	−0.726	0.474
Surgency	−388.600	1716	−0.226	0.822
Negative affectivity	160.300	1038.900	0.154	0.878
Education	284.800	1031	0.276	0.784
*R* ^2^ = 0.035, Δ*R* ^2^ = −0.132, *F* (5, 29) = 0.209, *p* = 0.956				
Regulatory/Orienting	−1270.500	1773.400	−0.716	0.480
Surgency	−388.700	1745.200	−0.223	0.825
Negative affectivity	164.300	1058.700	0.155	0.878
Education	291.800	1054.800	0.277	0.784
Gender	−141.600	2331.400	−0.061	0.952
Model 2: CTC				
*R* ^2^ = 0.239, Δ*R* ^2^ = 0.165, *F* (3, 31) = 3.244, *p* = 0.035				
Regulatory/Orienting	**−71.210**	**26.160**	**−2.722**	0.010*
Surgency	**−64.720**	**26.360**	**−2.456**	0.020*
Negative affectivity	−2.9630	18.860	−0.157	0.876
*R* ^2^ = 0.323, Δ*R* ^2^ = 0.233, *F* (4, 30) = 3.582, *p* = 0.017				
Regulatory/Orienting	**−61.116**	**26.278**	**−2.326**	0.022*
Surgency	**−64.040**	**26.550**	**−2.412**	0.028*
Negative affectivity	5.509	15.835	0.348	0.731
Education	−32.569	16.852	−1.933	0.063
*R* ^2^ = 0.341, Δ*R* ^2^ = 0.227, *F* (5, 29) = 2.999, *p* = 0.027				
Regulatory/Orienting	**−61.317**	**27.069**	**−2.265**	0.032*
Surgency	**−51.620**	**25.110**	**−2.056**	0.049*
Negative affectivity	5.596	16.222	0.345	0.733
Education	−34.201	17.017	−2.01	0.054
Gender	33.107	37.611	0.88	0.386
Model 2: CVC				
*R* ^2^ = 0.141, Δ*R* ^2^ = 0.057, *F* (3, 31) = 1.69, *p* = 0.189				
Regulatory/Orienting	−127.674	120.336	−1.061	0.297
Surgency	−149.380	117.991	−1.266	0.215
Negative affectivity	2.205	69.763	0.032	0.975
*R* ^2^ = 0.193, Δ*R* ^2^ = 0.0854, *F* (4, 30) = 1.793, *p* = 0.156				
Regulatory/Orienting	−127.120	118.540	−1.072	0.292
Surgency	−129.200	117.120	−1.103	0.279
Negative affectivity	26.620	70.910	0.375	0.710
Education	−98.210	70.370	−1.396	0.173
*R* ^2^ = 0.270, Δ*R* ^2^ = 0.145, *F* (5, 29) = 2.149, *p* = 0.088				
Regulatory/Orienting	−109.070	115.100	−0.948	0.351
Surgency	−129.020	113.270	−1.139	0.264
Negative affectivity	19.080	68.710	0.278	0.783
Education	−111.290	68.460	−1.626	0.115
Gender	265.440	151.320	1.754	0.089

*Note*: The single asterisk (*) and the bold font indicate *p* < 0.05.

The normality of the residuals was assessed using the Shapiro‐Wilk test. For the AWC regression, this was (*W* = 0.986, *p* = 0.945); for the CTC regression, *W* = 0.963, *p* = 0.285; and for the CVC regression, *W* = 0.954, *p* = 0.159. The results showed that neither temperament scores (*R* ^2^ = 0.032, Δ*R* ^2^ = −0.061, *F* (3, 31) = 0.344, *p* = 0.794), nor maternal education (*R* ^2^ = 0.035, Δ*R* ^2^ = −0.094, *F* (4, 30) = 0.267, *p* = 0.895) or infant gender (*R* ^2^ = 0.035, Δ*R* ^2^ = −0.132, *F* (5, 29) = 0.209, *p* = 0.956) explained significant variance in the data; they did not function as a predictor for AWC. The finding that neither AWC correlates with the infants' temperament nor that the infants' temperament predicts the AWC is in line with previous studies showing that AWC plays a subordinated role in infants' language development (Wang et al., 2020).

For CTC, temperament, more precisely Regulatory/Orienting and Surgency, precited CTC (*R* ^2^ = 0.239, Δ*R* ^2^ = 0.165, *F* (3, 31) = 3.244, *p* = 0.035), see Figure 1. Infants with higher scores in Surgency and Regulatory/Orienting had fewer communicative turns than infants with lower Surgency and Regulatory/Orienting scores. Adding maternal education (*R* ^2^ = 0.323, Δ*R* ^2^ = 0.233, *F* (4, 30) = 3.582, *p* = 0.017), and infant gender (*R* ^2^ = 0.341, Δ*R* ^2^ = 0.227, *F* (5, 29) = 2.999, *p* = 0.027), led to a higher percentage of variance explained by these variables however neither maternal education nor infant gender individually predicted CTC.

For CVC, the results showed that neither temperament (*R* ^2^ = 0.1406, Δ*R* ^2^ = 0.057, *F* (3, 31) = 1.690, *p* = 0.189) nor maternal education (*R* ^2^ = 0.193, Δ*R* ^2^ = 0.085, *F* (4, 30) = 1.793, *p* = 0.156) or infant gender (*R* ^2^ = 0.270, Δ*R* ^2^ = 0.145, *F* (5, 29) = 2.149, *p* = 0.088) explained significant amounts of variance of CVC. We suggest that the finding that neither CVC correlates with the infant temperament nor that the infant temperament predicts the CVC might be related to the infant's age. At the age of 4–5 months, infants are at the very early stages of their vocal activity, which may explain the lack of a clear relationship.

## 4 DISCUSSION

The aim of this study was to examine the relationship between infants' temperament and the quality and quantity of parental interaction. Quality was assessed through the expansion of maternal vowel space in IDS compared with that in their ADS, while quantity was measured by extracting the number of adult words, communicative turns, and infant vocalisations using the LENA system in a daylong recording. Infants' temperament traits were measured in terms of Surgency (reactivity to high levels of positive affect), Negative Affect (emotional distress level), and Regulatory/Orienting (Duration of Orienting, Cuddliness, and Soothability).

With respect to maternal IDS, our results show that even though mothers expand their vowel space during interactions, this expansion is not associated with any of the three aspects of infant temperament investigated here. One potential explanation for the lack of correlation between maternal vowel space expansion and infants' temperament may relate to the age of the infants. The infants, aged between 4 and 5 months, are at the onset of their perceptual attunement and babbling phases of speech perception and production aspects of linguistic development respectively (e.g., Werker, 2024). Given infants’ limited vocal activity at this stage, mothers may not receive clear cues from the infant's temperament that would influence their speech patterns thus obscuring any possible relationship between infant temperament and maternal vowel space. To examine this further a similar study with older infants with more advanced vocalisations, such as consistent babbling or early word articulations, would be useful.

An alternative explanation is that infant temperament interacts with maternal input in a complex manner. Our null results regarding the relationship between maternal input and infant temperament align with findings from Spinelli et al. (2018), who also observed no direct link between infant temperament and maternal input. However, their research revealed a complex interaction between maternal input—measured by lexical and syntactical variability—and infant temperament. They observed that infants with higher regulatory and orienting scores, along with mothers who employed a more diverse vocabulary and used more complex sentence structures at 6 and 9 months, had greater vocabulary at 18 months and greater mean length of utterances at 24 months. This suggests that the quality of maternal input is not directly tied to infant temperament, but rather serves as a catalyst for infant language development.

We consider a similar explanation for the absence of a link between vowel expansion and infant temperament in our study. A large body of research now supports the interpretation that maternal vowel space expansion has a facilitative role in infants' speech perception (Kalashnikova & Carreiras, 2022; Kalashnikova et al., 2018; Liu et al., 2003). For instance, Kalashnikova and Carreiras (2022) showed that infants whose mothers expanded their vowel space to a greater extent showed speech perception abilities indicative of more advanced perceptual attunement. This suggests that maternal vowel space expansion is linked to infants' speech perception development and plays a crucial role in attuning to native language speech contrasts (Kalashnikova & Carreiras, 2022).

We propose that maternal vowel space expansion is not directly influenced by infant temperament. Instead, as previous studies suggest, vowel space expansion serves a facilitative role in early linguistic development by providing clear and exaggerated speech sounds that aid in infants' speech perception. While infant temperament may not impact vowel space expansion at this early stage, it could become influential later in development. This influence may follow a quite complex pattern, as suggested by Spinelli et al. (2018), where interactions between maternal input and infant temperament predict later vocabulary size. Future studies investigating the impact of maternal vowel space expansion on infants' speech perception skills or vocabulary development may consider the influence of infant temperament on the development of these linguistic abilities.

Turning to the quantity of maternal speech, we identified a relationship between CTC—communicative turns, and the Surgency and Regulatory/Orienting scores (but not Negative Affectivity) of temperament. However, contrary to our initial hypothesis, in which we expected that infants with higher levels of Surgency would engage in more communicative turns, we found the opposite to be true: infants with lower levels of Surgency participated in more communicative turns. Similarly, infants with lower levels of Regulatory/Orienting exhibited more communicative turns.

What could explain the negative relationship between communicative turns and the temperament traits of Surgency and Regulatory/Orienting? One possible account of the findings arises from the way the LENA system calculates communicative turns and vocalisations. Firstly, the number of communicative turns could be reduced if the adult speaker was talking for a considerable period, which would allow less space for infant responses and thus reduce the count for turn‐taking. Secondly, the system counts a communicative turn only if a vocalisation is speechlike. The LENA system does not count laughter and crying as vocalisations, and consequently, those vocalisations are disregarded in the automatic counts. However, those types of emotional vocalisations may be crucial to understanding the relationship between infants' temperament and their communicative interactions with caregivers. More emotional vocal reactions such as laughter and giggles are evident in infants with higher levels of Surgency as they tend to respond to social stimuli with more positive affect, that is, smiles, enjoyment, and laughter (Ollas et al., 2020). Consequently, it is conceivable that some of the positive emotional vocal reactions exhibited by highly surgent infants here, as, for example, laughter, would not be recognised as a communicative turn by the LENA system. As a result, only infants with fewer non‐speechlike emotional vocalisations, potentially those with lower levels of Surgency, may display more communicative turns according to the LENA count. Further research is required to explore the relationship between infants' non‐speechlike vocal expressions, such as laughter and crying, their temperament and their communicative turn‐taking. Such data would allow us to draw more nuanced conclusions regarding the connection between temperament and parent‐infant interactions.

An alternative account of our finding is that infants with lower Surgency and Regulatory/Orienting scores engage in more frequent communicative turns during interactions with caregivers due to their lower attentiveness and responsiveness, which could lead caregivers to engage in more frequent but shorter turns to encourage and maintain engagement, thus fostering a pattern of more frequent back‐and‐forth interactions. Wolfe and Bell (2007) demonstrated that infants with low levels of Approach (similar to lower levels of Surgency in the current experiment) and lower Duration of Orienting (similar to lower levels of Regulation/Orienting in the current study) during infancy displayed better language skills in childhood. They suggested that language development of infants with higher levels of Approach (i.e., more extraverted) might be hindered by their outgoing behavior, while that of infants with lower Approach tendencies (i.e., more introverted) may benefit more from social interactions. Similarly, in accounting for our own data, it could be that infants high in Regulatory/Orienting create fewer interaction opportunities for parents as they sustain longer attention to objects and show a higher ability for self‐regulation. Number of communicative turns serves as a measure of these interactions and, interestingly, has previously been shown to be a better predictor of infant language development than the number of adult words (Dailey & Bergelson, 2023; Donnelly & Kidd, 2021; Wang et al., 2020). So, while two aspects of maternal speech alone, viz, maternal speech quality as reflected in vowel hyper‐scores, and maternal speech quantity as reflected in Adult Word Count, are not correlated with any aspect of infant temperament, the one aspect of quantity that reflects both sides of infant‐parent interactions, namely communicative turns, is correlated with the Surgency (Approach in Wolfe & Bell, 2007) and Regulatory/Orienting (Duration of Orienting in Wolfe and Bell) aspects of temperament. Hence, our findings may potentially provide the missing information required to bridge the gap in understanding why infants' temperament could predict later language outcomes. Specifically, this account suggests that a higher number of communicative turns, which may be more common in infants with lower levels of both Surgency and Regulatory/Orienting, could lead to better language outcomes, compatible with the findings of Wolfe and Bell (2007).

However, it is important to note that our study only analyzed 4‐month‐old infants and a direct link to later language outcomes based on infants' temperament and LENA output requires further investigation. Furthermore, the current study relied on vocal output automatically extracted by the LENA system. To validate these findings, we included a manual coding of 5‐min intervals and correlated the outcome from the manual measurements with the automatically extracted values. Even though the results showed high correlations between the outcome measures, future research should consider a broader sample of manual coding and automatically extracted measurements. In addition, future research should include additional methods of data collection and analysis, such as video coding of parent‐infant interactions. This approach would allow to examine variables such as the initiation of turns, the influence of non‐verbal cues like smiling, laughter, and crying, and the impact of infant temperament on these interactions. These more nuanced assessments of both caregiver‐infant interaction beyond automated measures could provide insights into the dynamics of early caregiver‐infant communication.

In summary, our findings suggest that infants' temperament is correlated with parent‐infant interactions. Infants with lower Surgency scores and lower Regulatory/Orienting scores engage in more turns than infants with higher scores. We suggest that infants with elevated Surgency levels might show a predisposition to approach social interactions, yet their outgoing behavior might potentially impede parental “replies” thus reducing the number of communicative turns. Conversely, more introverted infants (lower Surgency scores) may have benefited more from social interactions involving more communicative turns, due to their caregivers' efforts to encourage and maintain engagement. Moreover, infants displaying high levels of Regulatory/Orienting may present fewer opportunities for interaction with parents due to their lower activity levels, encompassing both positive and negative affective behaviors. Our findings suggest that infant temperament shapes how adults talk *with* infants (communicative turns), rather than how adults talk *to* infants (IDS, number of adult words) and thus underscores the important role of the infant in communication scenarios.

## AUTHOR CONTRIBUTIONS

**Antonia Götz**: Conceptualization; data curation; formal analysis; visualization; writing—original draft; writing—review and editing. **Eylem Altuntas**: Data curation; methodology; writing—original draft; writing—review and editing. **Marina Kalashnikova**: Conceptualization; funding acquisition; methodology; writing—original draft; writing—review and editing. **Catherine Best**: Conceptualization; funding acquisition; investigation; methodology; writing—original draft; writing—review and editing. **Denis Burnham**: Conceptualization; funding acquisition; methodology; project administration; writing—original draft; writing—review and editing.

## CONFLICT OF INTEREST STATEMENT

The authors declare no conflicts of interest with regard to the funding source for this study.

## Data Availability

Data available on request from the authors.
